# Perfluoroalkyl Chemicals and Asthma among Children 12–19 Years of Age: NHANES (1999–2008)

**DOI:** 10.1289/ehp.1306606

**Published:** 2014-06-06

**Authors:** Olivier Humblet, Ledif Grisell Diaz-Ramirez, John R. Balmes, Susan M. Pinney, Robert A. Hiatt

**Affiliations:** 1 Robert Wood Johnson Foundation Health and Society Scholars Program, Department of Psychiatry, University of California, San Francisco, San Francisco, California, USA; 2 University of California, Berkeley, School of Public Health, Berkeley, California, USA; 3 Department of Medicine, University of California, San Francisco, San Francisco, California, USA; 4 Department of Environmental Health, University of Cincinnati College of Medicine, Cincinnati, Ohio, USA; 5 Department of Epidemiology & Biostatistics, University of California, San Francisco, San Francisco, California, USA

## Abstract

Background: Perfluoroalkyl chemicals (PFCs) are a family of commonly used industrial chemicals whose persistence and ubiquity in human blood samples has led to concern about possible toxicity. Several animal studies and one recent human study have suggested a link between exposure to PFCs and asthma, although few epidemiologic studies have been conducted.

Objectives: We investigated children’s PFC serum concentrations and their associations with asthma-related outcomes.

Methods: We evaluated the association between serum concentrations of eight PFCs, including perfluorooctanoic acid (PFOA), perfluorooctane sulfonic acid (PFOS), perfluorononanoic acid (PFNA), and perfluorohexane sulfonic acid (PFHxS), with self-reported lifetime asthma, recent wheezing, and current asthma using data from participants 12–19 years of age from the 1999–2000 and 2003–2008 National Health and Nutrition Examination Surveys.

Results: In multivariable-adjusted models, PFOA was associated with higher odds of ever having received a diagnosis of asthma [odds ratio (OR) = 1.18; 95% CI: 1.01, 1.39 for a doubling in PFOA], whereas for PFOS there were inverse relationships with both asthma and wheezing (OR = 0.88; 95% CI: 0.74, 1.04, and OR = 0.83; 95% CI: 0.67, 1.02, respectively). The associations were attenuated after accounting for sampling weights. No associations were seen between the other PFCs and any outcome.

Conclusions: This cross-sectional study provides some evidence for associations between exposure to PFCs and asthma-related outcomes in children. The evidence is inconsistent, however, and prospective studies are needed.

Citation: Humblet O, Diaz-Ramirez LG, Balmes JR, Pinney SM, Hiatt RA. 2014. Perfluoroalkyl chemicals and asthma among children 12–19 years of age: NHANES (1999–2008). Environ Health Perspect 122:1129–1133; http://dx.doi.org/10.1289/ehp.1306606

## Introduction

Perfluoroalkyl chemicals (PFCs) are used in a wide variety of industrial and consumer-use applications, including textiles, food packaging, and nonstick cookware. Concerns about the toxicity of PFCs arose in the early 2000s after they were found to be ubiquitous and persistent in human blood samples worldwide (Lindstrom et al. 2011). One study estimated half-lives in humans of the two most common PFCs, perfluorooctane sulfonic acid (PFOS) and perfluorooctanoic acid (PFOA), at 5.4 and 3.8 years, respectively (Olsen et al. 2007); another study estimated a shorter half-life for PFOA of 2.3 years (Bartell et al. 2010). Various adverse health effects have been reported in animal studies, but relatively few human health studies have been conducted to date (Lindstrom et al. 2011; Steenland et al. 2010).

Asthma is a respiratory disease characterized by airway inflammation (Busse and Lemanske 2001), currently affecting 9.5% of U.S. children (Akinbami et al. 2012). Animal evidence indicates that exposures to PFOA and PFOS cause a variety of asthma-related outcomes including airway hyperresponsiveness and increased inflammation (Dong et al. 2011; Fairley et al. 2007; Singh et al. 2012; Zheng et al. 2011). The lungs were one of the main target organs of PFOA and PFOS in exposed rats (Cui et al. 2009). Furthermore, the hypothesis that PFCs are immunotoxic in humans is supported by the recent finding that concentrations of PFOA and PFOS in children’s serum at 5 years of age were associated with decreased serum concentrations of vaccine antibodies (Grandjean et al. 2012). Yet few studies had been published on PFCs and asthma in humans until a recent article (Dong et al. 2013) reported positive associations of PFC serum concentrations with asthma, asthma severity, and immunological markers in Taiwanese children.

We seek to add to this sparse literature using cross-sectional data from the National Health and Nutrition Examination Survey (NHANES). PFCs were measured in serum of participants ≥ 12 years of age, beginning in 1999. In this analysis we focused on PFCs among children 12–19 years, and the prevalence of self-reported wheezing and physician-diagnosed asthma. The study was restricted to children because asthma is primarily an early-onset disease, and thus measures of PFCs in children are more reflective of exposure prior to disease.

## Materials and Methods

*Study population*. The National Health and Nutrition Examination Survey (NHANES) is conducted by the U.S. Centers for Disease Control and Prevention (CDC) and designed to assess health and nutrition in a nationally representative sample of the civilian, noninstitutionalized U.S. population (CDC 2014). The survey consists of interviews and physical examinations of approximately 5,000 persons each year. Data are released in 2-year cycles. The present study used data from four cycles: 1999–2000, 2003–2004, 2005–2006, and 2007–2008. Eligibility criteria for this study consisted of being 12–19 years of age, having a measured serum PFC concentration, and having data on the asthma-related outcomes of interest.

NHANES participants provided informed consent before participation in the NHANES study. This analysis was covered by the original consent, and was determined to be exempt from institutional review board review.

*Exposure assessment*. Sera were analyzed for 12 PFCs among a subsample of participants ≥ 12 years of age from NHANES 1999–2000 and 2003–2008. Details regarding the analytical procedures have been published previously (Calafat et al. 2007; Kato et al. 2011a; Kuklenyik et al. 2005). For PFC concentrations below the limit of detection (LOD), a value equal to LOD divided by the square root of 2 was substituted.

Of the 12 PFCs analyzed in NHANES, our analyses focused on the four PFCs that were detectable in > 96% of individuals in the eligible age range: PFOA, PFOS, perfluorohexane sulfonic acid (PFHxS), and perfluorononanoic acid (PFNA) (Kato et al. 2011b). Four additional PFCs were analyzed as binary variables in exploratory analyses because of moderate proportions of nondetects (i.e., 30–75% not detected over the four survey cycles): 2-(*N*-ethyl-perfluorooctane sulfonamido) acetate (EPAH), 2-(*N*-methyl-perfluorooctane sulfonamido) acetate (MPAH), perfluorodecanoic acid (PFDE), and perfluorooctane sulfonamide (PFSA). The remaining four PFCs were not analyzed because they were detected in < 25% of the samples in all survey cycles.

*Asthma assessment*. The three self-reported asthma-related outcomes used were ever having received a diagnosis of asthma from a doctor or other health professional (ever asthma), wheezing or whistling in the chest in the preceding 12 months (wheezing in past year), and answering yes to the question “do you still have asthma” (current asthma). “Ever asthma” was selected because of the long half-life of PFCs in the body, whereas “wheezing in past year” and “current asthma” were chosen to assess the sensitivity of recent respiratory symptoms to serum PFC concentrations assessed cross-sectionally. For current asthma, the comparison group was people who had never received a diagnosis of asthma.

*Statistical analysis*. All statistical tests used α = 0.05 as the threshold for statistical significance. All analyses were conducted using SAS 9.3 (SAS Institute Inc., Cary, NC). Proc Surveylogistic was used for all analyses unless otherwise specified. NHANES sampling clusters and strata were incorporated into all the analyses that used Proc Surveylogistic (National Center for Health Statistics 2014).

The final regression models included the following covariates in addition to each PFC variable: NHANES wave (1999–2000, 2003–2004, 2005–2006, 2007–2008); age (12–13, 14–15, 16–17, 18–19 years); sex; race/ethnicity (Mexican American–U.S. born, Mexican American–Mexican born, other Hispanic, non-Hispanic white/other race, non-Hispanic black); poverty income ratio (obtained by dividing family income by the poverty guidelines specific to family size, year, and state; < 1, 1–2, ≥ 2); ever cigarette smoking by the child; and health insurance (none, government, private). Variables were considered for inclusion based on standard causal criteria (Hernán et al. 2002). Current household smoking was excluded for not being a significant predictor of any asthma outcome. Body mass index (BMI) was excluded in the primary analyses (but included in sensitivity analyses) because it is a potential intermediate of an association between PFCs and asthma. Country of origin was incorporated into the race/ethnicity variable for Mexican Americans because it has been shown to be linked with PFC exposure (Nelson et al. 2012), as well as asthma prevalence (Subramanian et al. 2009). Observations with complete data for the final covariates were included (85.9% of the 2,186 children with data on asthma and PFCs). Multiple imputation was conducted in a sensitivity analysis to check if the results were affected by the missing data (Berglund 2010).

Once the multivariable models were chosen, natural cubic splines with 3 degrees of freedom were used in Proc Logistic to visually assess the linearity of the dose–response relationship with each asthma outcome for both untransformed and natural log (ln)–transformed PFC variables. Three multivariable models (ln-linear, linear, and tertiles) were used to analyze each exposure–outcome pair. The Akaike information criterion (AIC) was used to compare model fit. Either the ln-linear or linear model was best-fitting, depending on the association, although their AICs were similar. Ln-transformed PFCs were chosen for sensitivity analyses because they were more linearly associated with the asthma outcomes. Odds ratios (ORs) from the ln-linear model are presented for a doubling of the serum PFC concentration, calculated using the formula OR = exp[β⊇× ln(2)], where β is the regression coefficient for a ln-transformed PFC variable. The presence of effect modification by sex and race/ethnicity (i.e., white vs. nonwhite) was explored using multiplicative interaction terms for the PFCs with evidence of main effects on the asthma outcomes.

NHANES data are collected using a complex, multistage, probability sampling design to select participants representative of the civilian, noninstitutionalized U.S. population. Oversampling of certain population subgroups is done to increase the reliability and precision of health status indicator estimates for these groups. Oversampled subgroups include African Americans, Mexican Americans, low-income white Americans (beginning in 2000), and adolescents 12–19 years of age. A sample weight is assigned to each sample person, reflecting the unequal probability of selection, nonresponse adjustment, and adjustment to independent population controls. Although incorporating the sample weights into the analyses is recommended when the goal is to obtain unbiased national estimates, this also has the limitation of decreasing the statistical power of the study. It has been suggested that the optimal balance of power and validity is achieved by conducting unweighted analyses that instead adjust for the variables used in defining the sample weights (Korn and Graubard 1991). Our primary analyses used the latter approach, and we incorporated the sample weights in sensitivity analyses.

An additional sensitivity analysis was conducted because the ln-transformation of PFC concentrations caused a few of the PFC concentrations that were close to 0 to have ln-transformed values well below 0. To assess whether this was affecting the model coefficients, we created an alternative transformation of the PFC concentrations that was equal to ln(PFC concentration +1) (Mosteler and Tukey 1977). Results using these new variables were compared with the ln-transformed results in all analyses. Finally, the four PFCs with moderate quantities of nondetects (EPAH, MPAH, PFDE, and PFSA) were analyzed as binary variables, above and below the LOD.

## Results

We evaluated the association between PFCs and self-report of both asthma and wheezing among 1,877 children 12–19 years of age (mean ± SD, 15.4 ± 2.3). Of these, 318 (16.9%) were ever diagnosed with asthma, 217 (11.6%) reported wheezing in the preceding 12 months, and 191 (10.2%) reported current asthma. The NHANES children with measured PFC serum concentrations were similar in their asthma prevalence and demographics to children of the same age without PFC measurements (data not shown). The serum concentrations of the four primary PFCs were generally moderately correlated. The highest Spearman coefficient was 0.68, for PFOA and PFOS. All other correlations between these four PFCs ranged from 0.18 to 0.49, except for the coefficient for PFOS and PFNA, which was 0.05.

In univariate analyses (Table 1), similar trends were seen for the prevalence of all three outcomes across categories of race/ethnicity (lowest for Mexican-born Mexican Americans) and health insurance (highest for private insurance, lowest for no insurance). Asthma prevalence did not increase significantly over this time period, consistent with evidence suggesting relatively stable asthma rates in the United States since the late 1990s (Moorman et al. 2007). PFC concentrations and their predictors within this sample have been previously described (Kato et al. 2011b; Nelson et al. 2012).

**Table 1:** Prevalence (%) of asthma and wheeze by participant characteristics, NHANES 1999–2008, children 12–19 years of age (*n* = 1,877).

Characteristic	All	Ever asthma	Wheezing in past year	Current asthma
Overall		16.9	11.6	10.2
Wave of NHANES data collection
1999–2000	24.6	13.9	10.8	8.0
2003–2004	29.6	17.5	13.3	11.0
2005–2006	29.9	18.9	11.2	11.0
2007–2008	15.8	17.2	10.1	10.5
*p*-Value		0.19	0.46	0.36
Age (years)
12–13	26.7	17.2	12.0	11.4
14–15	23.5	18.4	12.5	10.5
16–17	24.5	17.8	10.2	11.3
18–19	25.3	14.5	11.6	7.6
*p*-Value		0.41	0.74	0.17
Sex
Male	52.5	18.5	12.9	10.6
Female	47.5	15.3	10.1	9.8
*p*-Value		0.07	0.06	0.56
Race/ethnicity
Mexican American (U.S. born)	24.2	15.6	12.8	10.2
Mexican American (Mexican born)	9.5	5.1	5.1	3.9
Other Hispanic	6.0	21.2	10.6	12.4
Non-Hispanic white/other race	30.3	19.3	12.8	10.1
Non-Hispanic black	30.0	18.5	11.6	11.9
*p*-Value		0.0001	0.06	0.04
Poverty income ratio
< 1	33.4	16.0	11.3	9.3
1 to < 2	27.5	15.7	10.3	9.1
≥ 2	39.1	18.7	12.7	11.7
*p*-Value		0.27	0.40	0.21
Ever smoking by child
Yes	40.3	18.0	13.0	10.3
No	59.7	16.2	10.6	10.1
*p*-Value		0.32	0.12	0.89
Health insurance
None	22.9	11.7	9.3	6.8
Government	31.1	17.0	11.5	10.7
Private	46.1	19.5	12.7	11.6
*p*-Value		0.0018	0.20	0.02
Prevalences do not incorporate NHANES survey weights.				

Table 2 compares the concentrations of the four primary PFCs in children with and without each of the three asthma outcomes. In every case, the median PFC concentration was higher among the children with asthma or wheeze, except for PFOS versus current asthma. However, only for PFOA and PFNA versus ever asthma was the *p*-value for the difference less than or close to 0.05.

**Table 2:** Serum PFC concentrations (ng/mL) in children with and without asthma or wheezing.

Variable	PFOA	PFOS	PFNA	PFHxS
Never asthma (*n *= 1,559)
Median (IQR)	4.0 (2.8, 5.4)	16.8 (10.8, 26.2)	0.8 (0.5, 1.2)	2.0 (1.0, 4.1)
5th–95th percentile	1.5–8.2	5.7–45.6	0.2–2.4	0.4–11.9
Percent > LOD	99.8	100.0	98.5	98.5
Ever asthma (*n *= 318)
Median (IQR)	4.3 (3.1, 5.7)	17.0 (10.8, 25.8)	0.9 (0.6, 1.2)	2.2 (1.1, 3.9)
5th–95th percentile	1.9–9.8	5.6–43.2	0.2–2.6	0.6–12.3
Percent > LOD	100.0	100.0	98.1	97.8
*p*-Value^*a*^	0.034	0.885	0.053	0.414
No wheezing past 12 months (*n *= 1,660)
Median (IQR)	4.0 (2.9, 5.5)	16.8 (10.8, 26.2)	0.8 (0.5, 1.2)	2.0 (1.1, 4.2)
5th–95th percentile	1.6–8.4	5.8–44.6	0.2–2.4	0.4–11.7
Percent > LOD	99.8	100.0	98.4	98.3
Wheezing past 12 months (*n *= 217)
Median (IQR)	4.4 (2.9, 5.6)	17.2 (10.9, 25.4)	0.8 (0.5, 1.2)	2.2 (1.1, 3.6)
5th–95th percentile	1.7–8.6	4.8–47.7	0.2–2.3	0.5–14.4
Percent > LOD	100.0	100.0	99.1	99.1
*p*-Value^*a*^	0.188	0.860	0.702	0.697
No current asthma (*n *= 1,559)
Median (IQR)	4.0 (2.8, 5.4)	16.8 (10.8, 26.2)	0.8 (0.5, 1.2)	2.0 (1.0, 4.1)
5th–95th percentile	1.5–8.2	5.7–45.6	0.2–2.4	0.4–11.9
Percent > LOD	99.8	100.0	98.5	98.5
Current asthma (*n *= 191)
Median (IQR)	4.2 (2.9, 5.6)	16.7 (10.3, 25.3)	0.9 (0.5, 1.3)	2.1 (1.0, 3.9)
5th–95th percentile	1.9–9.0	5.0–45.6	0.3–2.4	0.5–14.4
Percent > LOD	100.0	100.0	99.0	97.4
*p*-Value^*a*^	0.231	0.642	0.121	0.638
IQR, interquartile range. Values in table do not incorporate NHANES survey weights. ^***a***^Wilcoxon rank-sum test to compare the PFC serum concentrations between children with and without asthma or wheezing.				

All multivariable exposure–outcome relationships were monotonic and approximately linear on the natural log scale over the central part of the exposure distribution (see Supplemental Material, Figures S1–S4). A significant increase in the odds of ever asthma was seen for an increase in linear or ln-linear serum PFOA concentration (Table 3), whereas serum PFOS was associated with lower odds of ever asthma and wheeze, albeit with 95% CIs that included the null value. The results were unchanged when using an alternate ln-transformation of the PFC exposure variables [i.e., ln(PFC + 1)] (data not shown). Effect modification by either sex or race/ethnicity (white vs. nonwhite) was tested in models using PFOA and PFOS as the exposure variables. No interactions with *p*-values < 0.10 were seen (data not shown). Adjusting for BMI (a potential intermediate) in sensitivity analyses did not meaningfully change the PFC coefficients (data not shown).

**Table 3:** Serum PFC ORs (95% CIs) for asthma and wheeze among children 12–19 years of age (*n* = 1,877).

Model	Ever asthma			Wheeze			Current asthma		
	AIC	OR (95% CI)	*p*-Value	AIC	OR (95% CI)	*p*-Value	AIC	OR (95% CI)	*p*-Value
PFOA
Ln-linear^*a*^	1696.2	1.18 (1.01, 1.39)	0.04	1357.5	1.00 (0.80, 1.23)	0.98	1244.6	1.12 (0.92, 1.36)	0.26
Linear	1696.1	1.06 (1.00, 1.11)	0.03	1357.5	1.01 (0.94, 1.07)	0.87	1244.8	1.03 (0.97, 1.10)	0.30
Tertile 1		1.00	Referent		1.00	Referent		1.00	Referent
Tertile 2	1698.3	1.06 (0.89, 1.27)	0.52	1358.9	0.95 (0.76, 1.19)	0.66	1245.4	0.89 (0.70, 1.12)	0.33
Tertile 3		1.11 (0.94, 1.31	0.20		1.08 (0.86, 1.36)	0.49		1.18 (0.90, 1.53)	0.23
PFOS
Ln-linear^*a*^	1697.0	0.88 (0.74, 1.04)	0.13	1354.0	0.83 (0.67, 1.02)	0.08	1244.1	0.88 (0.72, 1.09)	0.24
Linear	1696.7	0.99 (0.98, 1.00)	0.07	1356.6	0.99 (0.98, 1.01)	0.37	1244.8	0.99 (0.98, 1.01)	0.34
Tertile 1		1.00	Referent		1.00	Referent		1.00	Referent
Tertile 2	1701.1	0.96 (0.77, 1.19)	0.70	1357.9	0.93 (0.73, 1.18)	0.53	1246.2	0.88 (0.69, 1.13)	0.31
Tertile 3		1.01 (0.80, 1.27)	0.95		0.93 (0.70, 1.24)	0.62		1.06 (0.80, 1.41)	0.69
PFNA
Ln-linear^*a*^	1699.3	0.99 (0.88, 1.12)	0.92	1357.5	0.99 (0.84, 1.18)	0.94	1245.6	1.00 (0.76, 1.33)	0.97
Linear	1699.0	1.05 (0.89, 1.23)	0.56	1357.5	1.00 (0.81, 1.22)	0.96	1245.5	1.02 (0.81, 1.30)	0.86
Tertile 1		1.00	Referent		1.00	Referent		1.00	Referent
Tertile 2	1700.9	0.95 (0.80, 1.12)	0.52	1358.9	1.08 (0.89, 1.32)	0.43	1246.8	0.90 (0.71, 1.14)	0.40
Tertile 3		0.99 (0.84, 1.17)	0.95		0.97 (0.75, 1.25)	0.80		1.05 (0.82, 1.33)	0.71
PFHS
Ln-linear^*a*^	1699.0	0.98 (0.88, 1.08)	0.66	1357.5	0.99 (0.90, 1.10)	0.92	1245.6	1.00 (0.82, 1.22)	0.99
Linear	1698.8	0.99 (0.96, 1.02)	0.52	1357.5	1.00 (0.97, 1.03)	0.98	1245.4	1.01 (0.98, 1.03)	0.71
Tertile 1		1.00	Referent		1.00	Referent		1.00	Referent
Tertile 2	1700.3	1.07 (0.89, 1.30)	0.46	1356.9	1.16 (0.98, 1.37)	0.08	1246.9	0.94 (0.75, 1.16)	0.54
Tertile 3		0.92 (0.74, 1.14)	0.44		0.87 (0.71, 1.06)	0.17		0.98 (0.76, 1.28)	0.91
All models were adjusted for sex, smoking, age, race/ethnicity, survey cycle, poverty income ratio, and health insurance. Tertiles are shown by ascending concentration. ^***a***^ORs shown for a doubling of PFC concentration.									

When the NHANES survey weights were incorporated into the ln-linear regression models (see Supplemental Material, Table S1), the association between PFOA and ever asthma was attenuated (OR = 1.11; 95% CI: 0.87, 1.42) compared with the results in Table 3. As expected, all standard errors increased after incorporating the weights.

After multiple imputation of missing data in ln-linear models (see Supplemental Material, Table S2), the association between PFOA and ever asthma was again attenuated (OR = 1.13; 95% CI: 0.99, 1.29) compared with the results in Table 3. However, the overall inference remained similar for this association (*p* = 0.06) due to a decrease in the magnitude of the standard error.

EPAH, MPAH, PFDE, and PFSA concentrations were analyzed as binary variables (i.e., above and below the LOD). The effect estimates were imprecise and mostly close to the null value (see Supplemental Material, Table S3).

## Discussion

This cross-sectional analysis among U.S. children 12–19 years of age is one of the first to evaluate the association between serum PFC concentrations and asthma in children. This question has public health relevance because of the high prevalence of asthma and the ubiquitous nature of PFC exposures in this country. We estimated a positive association between serum PFOA concentrations and ever having been diagnosed with asthma (but not self-reported wheeze in the past year or current asthma), and negative associations of serum PFOS concentrations with ever being diagnosed with asthma and with wheezing in the past year, based on multivariable adjusted models. The magnitude of the PFOA–asthma association was attenuated after incorporating the NHANES survey weights so as to make the results representative of the U.S. population.

The first published study, to our knowledge, to assess the PFC–asthma association in children (Dong et al. 2013) recently reported positive associations of asthma, asthma severity, and altered immunological markers in Taiwanese children (*n* = 231 asthma cases and 225 controls) with PFOA, PFOS, and other PFCs. However, in this case–control study the asthma cases were recruited differently from the controls (in hospitals vs. in schools), leading to potential selection bias or confounding if controls do not represent the population that gave rise to the cases. This limitation does not apply to their finding that asthma severity among the cases was also associated with PFC concentrations. Other studies have reported mixed results regarding *in utero* PFC exposure in relation to asthma and allergic outcomes, although their applicability to childhood exposure is unclear. Among 244 Taiwanese children (Wang et al. 2011), cord blood concentrations of both PFOA and PFOS were associated with higher cord blood concentration of immunoglobulin E (IgE), a marker of predisposition to allergy. However a study in Japan (*n* = 343 infants, 33 with wheeze) reported that cord blood IgE levels were lower in association with high maternal PFOA levels among female infants, whereas there was no evidence of a relationship between maternal PFOS and PFOA levels and wheeze (Okada et al. 2012). Unpublished findings from the C8 Study Panel indicated that asthma prevalence among children (*n* = 878; 185 reporting ever asthma) was inversely associated with the quartile of maternal prenatal PFOA concentration (C8 Science Panel 2012). In contrast, animal evidence (DeWitt et al. 2012) has linked exposure to PFOA with airway hyperresponsiveness (Fairley et al. 2007) and allergic inflammation (Singh et al. 2012), and exposure to PFOS with an immune shift toward an allergic T-helper (Th) 2 phenotype (Dong et al. 2011; Zheng et al. 2011).

The present study has several limitations in its analysis and study design. First, the primary analyses assessed three disease outcomes in relation to eight PFCs. Conducting this number of comparisons increases the possibility of discovering significant associations by chance. Second, due to the cross-sectional design, the exposure biomarkers for these 12- to 19-year-olds were collected after asthma incidence had occurred, leading to possible exposure misclassification. Of the asthmatic children, 83% were diagnosed before 12 years, and 52% before 5 years. The extent to which the measured PFC concentrations provide accurate estimates of the etiologically relevant exposures depends on the similarity between these two values within individuals, due to the multi-year half-life of PFCs and/or the presence of ongoing PFC exposure. The extent of this similarity is unknown, but substantial exposure misclassification is likely. Third, because exposure and outcome are measured simultaneously, it is impossible to establish whether the exposure preceded the outcome (Fei et al. 2012). Although it is theoretically possible that asthma might affect the excretion of PFCs, we know of no plausible mechanism through which this might occur. Another hypothetical possibility is that PFCs could be incorporated into asthma medications, because recent publications have explored the technical feasibility of doing so (Butz et al. 2002; Lehmler 2007). We have been unable to find publicly available information regarding whether this is in fact the case. Fourth, no information was available in NHANES on the duration of breastfeeding for children of this age, even though this is a potential confounder that influences the accumulation of PFCs (Kärrman et al. 2007) and possibly also the incidence of asthma (Brew et al. 2011; Dogaru et al. 2012; Matheson et al. 2012).

The outcomes in this study were the self-report of physician-diagnosed asthma, wheezing symptoms, and current asthma. All are subject to potential errors in reporting by the study participants. Physician-diagnosed asthma could additionally be affected by variability in physicians’ diagnostic criteria or in access to health care (e.g., in the present study, the children with private health insurance were almost twice as likely to have ever been told they had asthma). Although there is no single generally accepted operational definition of asthma, the self-report of a physician’s diagnosis of asthma is among the diagnostic questions with both the highest specificity and highest reliability (Toren et al. 1993).

The present study is, to our knowledge, the only investigation of asthma in children related to PFC exposures in the United States and adds to a sparse worldwide literature on this topic. Similar to Dong et al. (2013), we estimated a significantly higher prevalence of asthma among children in association with a doubling of serum PFOA concentrations, albeit of a weaker magnitude. We believe this relationship deserves further exploration. However, caution should be taken in interpreting the strength of our findings, in light of this study’s mixed results and methodological limitations. Although PFOA concentrations were positively associated with asthma, PFOS was negatively associated with asthma outcomes. These findings contrast with other asthma-related human and experimental studies that have not reported opposing associations for PFOA and PFOS.

In summary, the findings from the present study highlight the need for additional research to evaluate the association of PFCs, especially PFOA and PFOS, with asthma outcomes in prospective studies of children.

## Supplemental Material

(1.8 MB) PDF
