# Supplementary material for: Perfluoroalkyl Chemicals and Asthma among Children 12–19 Years of Age: NHANES (1999–2008)
Source: Environ Health Perspect. 2014 Jun 6;122(10):1129–33. doi: 10.1289/ehp.1306606 (PMC4181915; doi:10.1289/ehp.1306606)
Supplement: (1.8 MB) PDF [file ehp.1306606.s001.508.pdf]

Supplemental Material

Perfluoroalkyl Chemicals and Asthma among Children 12–19 Years of Age: NHANES (1999–2008)

Olivier Humblet, Ledif Grisell Diaz-Ramirez, John R. Balmes, Susan M. Pinney, and Robert A. Hiatt

Table of Contents **Page**

Figure S1. Predicted probabilities of ‘Ever Asthma’, ‘Wheeze in Past Year’, and ‘Current Asthma’ by ln-transformed serum PFOA concentration 2

Figure S2. Predicted Probabilities of ‘Ever Asthma’, ‘Wheeze in Past Year’, and ‘Current Asthma’ by ln-transformed serum PFOS concentration 3

Figure S3. Predicted Probabilities of ‘Ever Asthma’, ‘Wheeze in Past Year’, and ‘Current Asthma’ by ln-transformed serum PFNA concentration 4

Figure S4. Predicted Probabilities of ‘Ever Asthma’, ‘Wheeze in Past Year’, and ‘Current Asthma’ by ln-transformed serum PFHxS concentration 5

Table S1. Odds Ratios, after incorporating NHANES survey weights, of 'Ever Asthma', 'Wheeze in Past Year', and ‘Current Asthma’ for a doubling of serum PFC concentrations among children ages 12-19 6

Table S2. Odds Ratios, after multiple imputation of missing values, of 'Ever Asthma', 'Wheeze in Past Year', and ‘Current Asthma’ for a doubling of serum PFC concentrations among children ages 12-19 7

Table S3. Odds Ratios of 'Ever Asthma', 'Wheeze in Past Year', and ‘Current Asthma’ for values above the limit of detection of EPAH, MPAH, PFDE, and PFSA among children ages 12-19 8

Reference 9

Figure S1. Predicted probabilities of 'Ever Asthma', 'Wheeze in Past Year', and 'Current Asthma' by ln-transformed serum PFOA concentration. All models adjusted for sex, smoking, age, race/ethnicity, survey cycle, poverty income ratio, and health insurance.

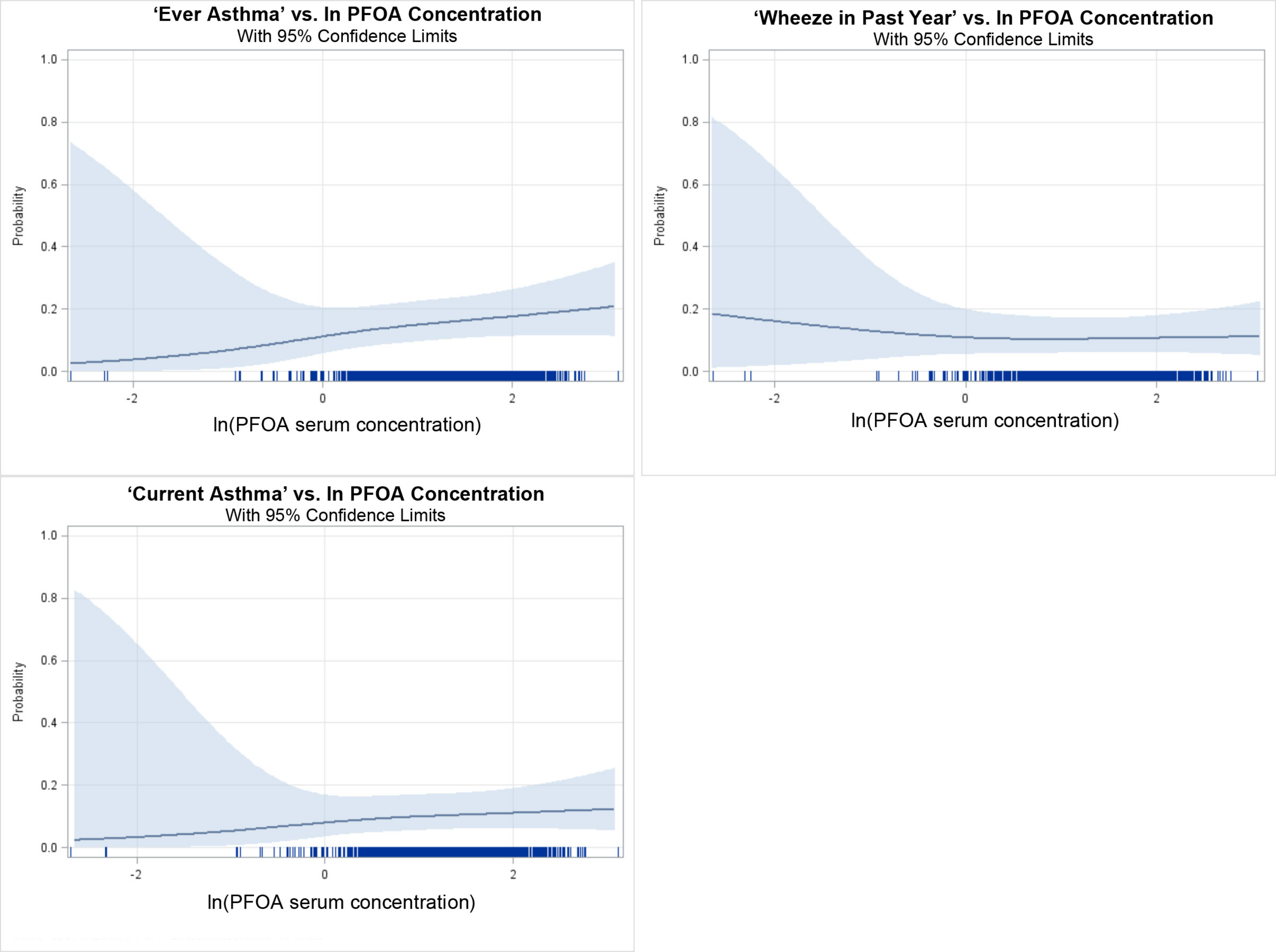

Figure S2. Predicted Probabilities of ‘Ever Asthma’, ‘Wheeze in Past Year’, and ‘Current Asthma’ by ln-transformed serum PFOS concentration. All models adjusted for sex, smoking, age, race/ethnicity, survey cycle, poverty income ratio, and health insurance.

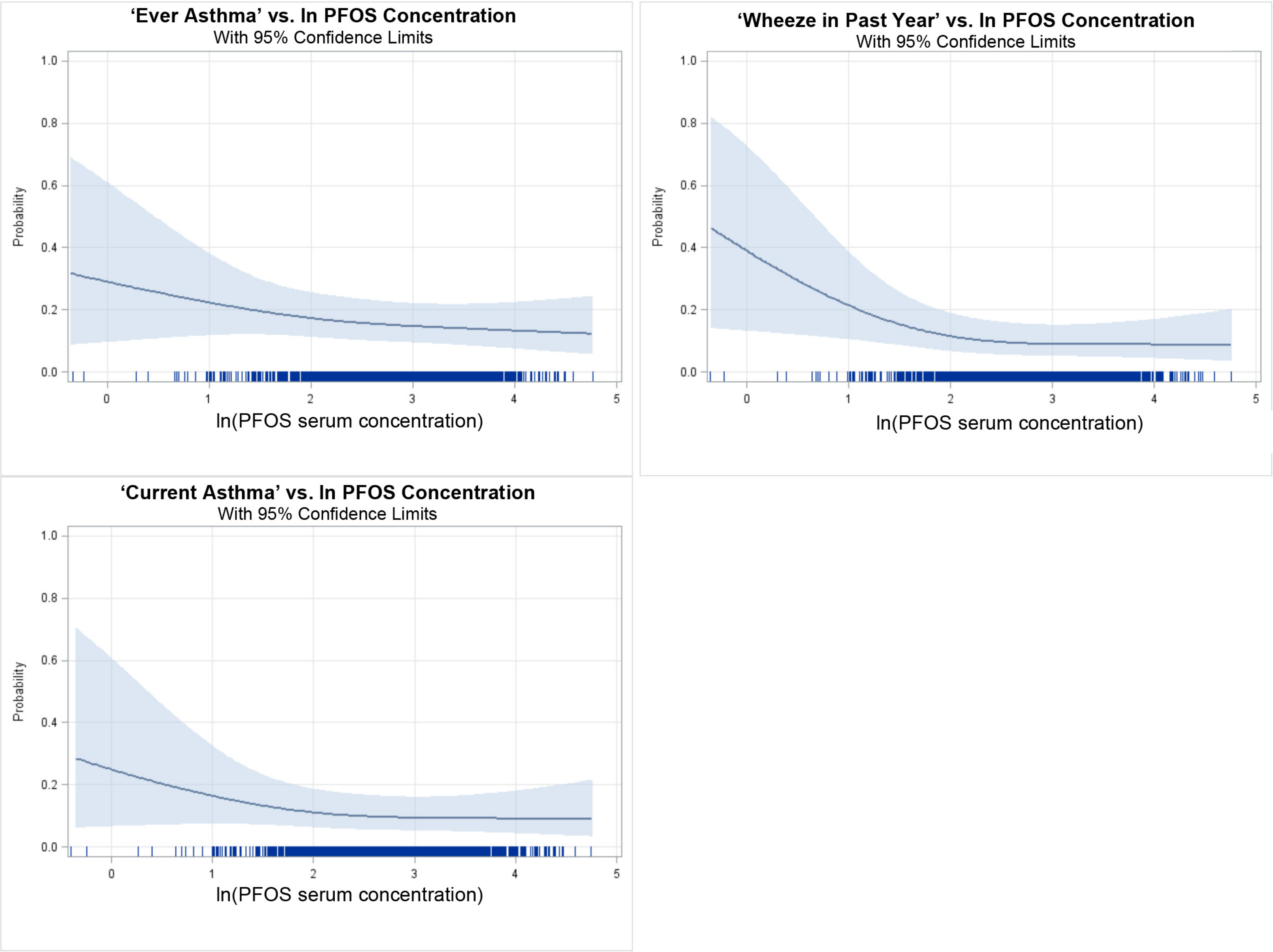

Figure S3. Predicted Probabilities of 'Ever Asthma', 'Wheeze in Past Year', and 'Current Asthma' by ln-transformed serum PFNA concentration. All models adjusted for sex, smoking, age, race/ethnicity, survey cycle, poverty income ratio, and health insurance.

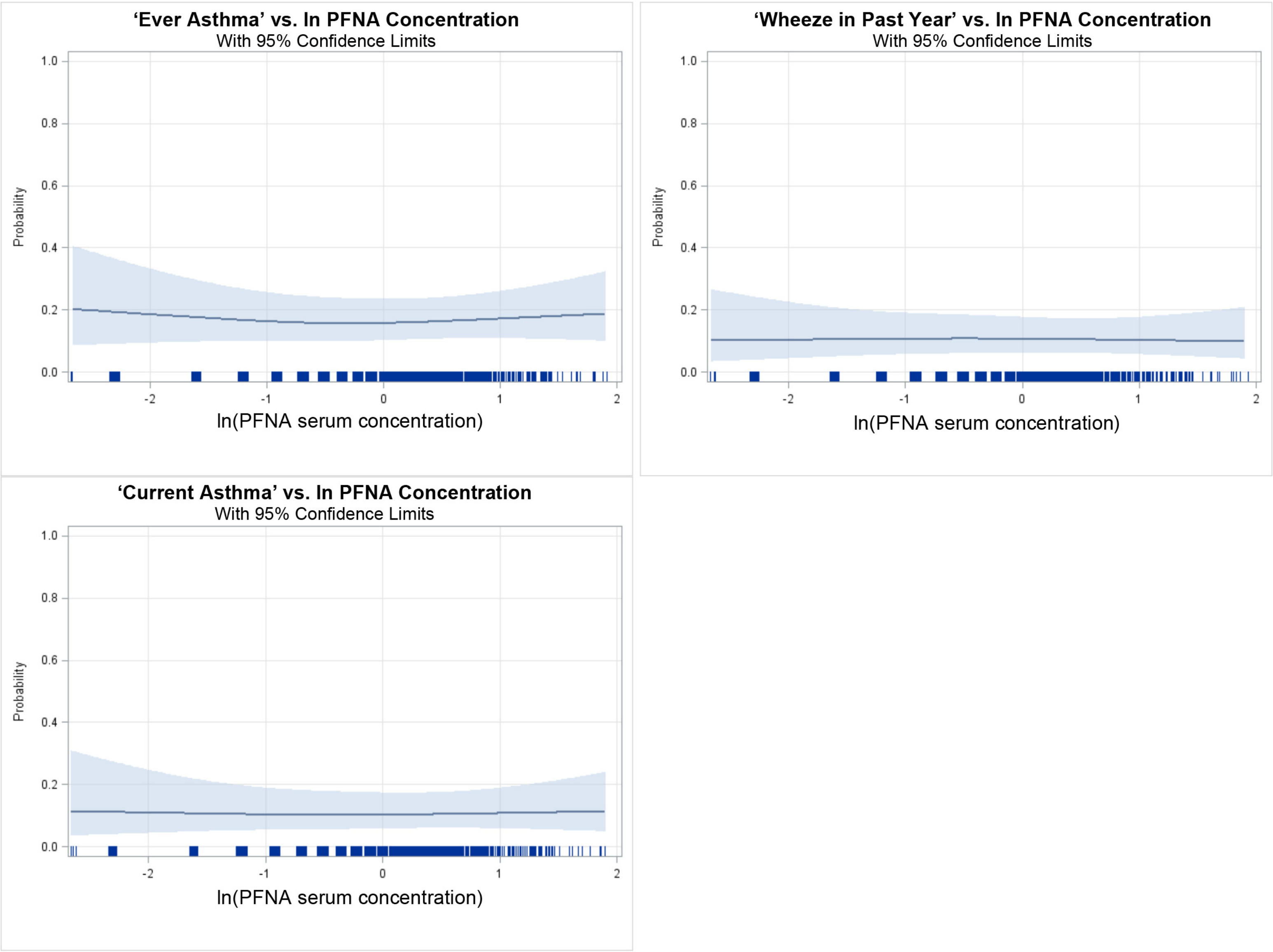

Figure S4. Predicted Probabilities of 'Ever Asthma', 'Wheeze in Past Year', and 'Current Asthma' by ln-transformed serum PFHxS concentration. All models adjusted for sex, smoking, age, race/ethnicity, survey cycle, poverty income ratio, and health insurance.

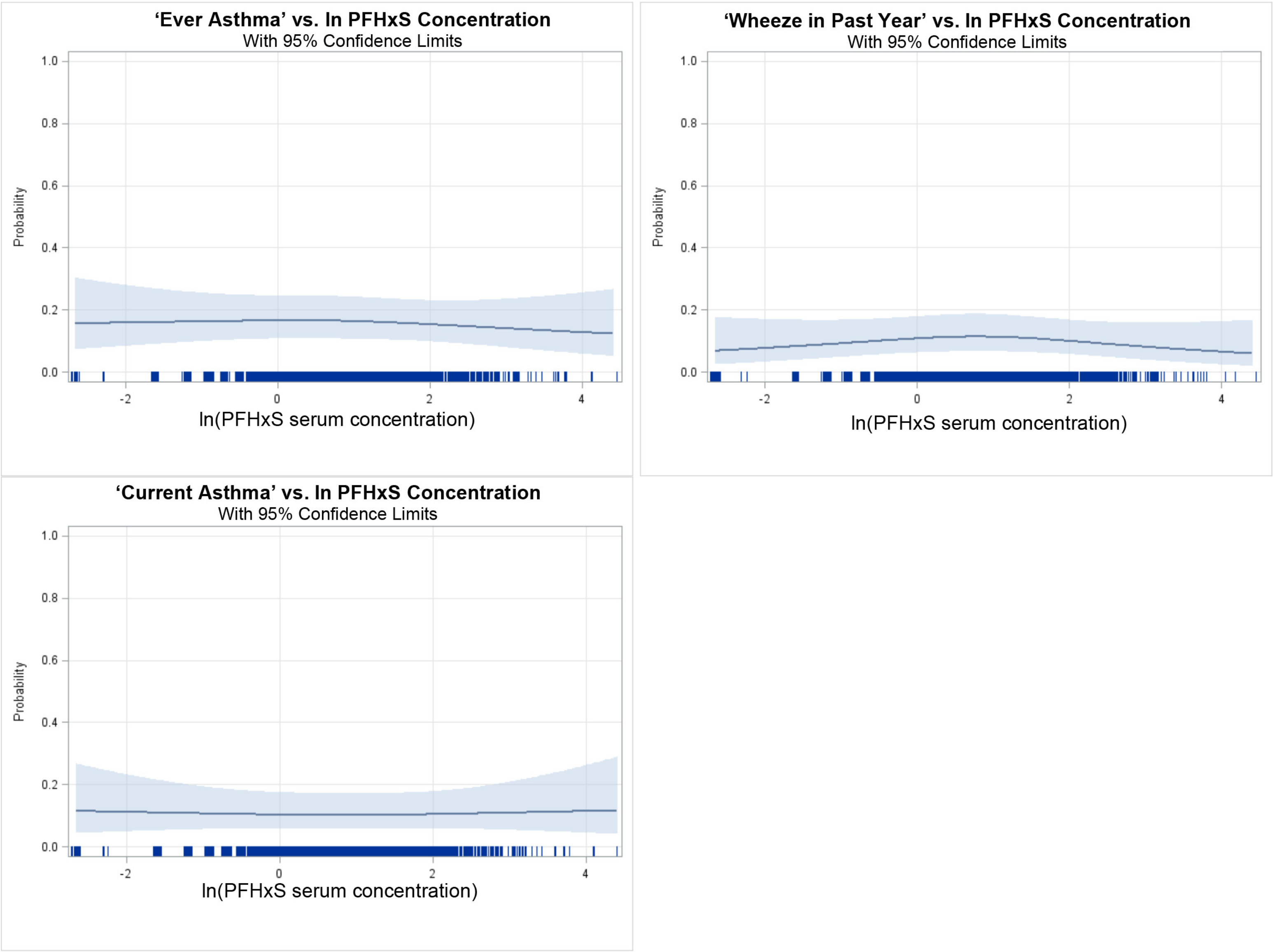

Table S1. Odds Ratios, after incorporating NHANES survey weights, of 'Ever Asthma', 'Wheeze in Past Year', and ‘Current Asthma’ for a doubling of serum PFC concentrations among children ages 12-19 (n = 1,877).

PFC	Ever Asthma: OR (CI)	Ever Asthma: *p*-value	Wheeze: OR (CI)	Wheeze: *p*-value	Current Asthma: OR (CI)	Current Asthma: *p*-value
PFOA	1.11 (0.87, 1.42)	0.42	1.14 (0.82, 1.58)	0.43	1.15 (0.86, 1.55)	0.35
PFOS	0.84 (0.66, 1.08)	0.17	0.87 (0.67, 1.13)	0.29	0.91 (0.68, 1.21)	0.51
PFNA	0.99 (0.84, 1.16)	0.89	1.03 (0.84, 1.26)	0.78	0.99 (0.80, 1.23)	0.95
PFHS	0.92 (0.79, 1.06)	0.25	0.92 (0.81, 1.06)	0.25	0.95 (0.76, 1.20)	0.67

*p<0.05. All models adjusted for sex, smoking, age, race/ethnicity, survey cycle, poverty income ratio, and health insurance.

Table S2. Odds Ratios, after multiple imputation of missing values, of 'Ever Asthma', 'Wheeze in Past Year', and ‘Current Asthma’ for a doubling of serum PFC concentrations among children ages 12-19 (n = 2,186).

PFC	Ever Asthma: OR (CI)	Ever Asthma: *p*-value	Wheeze: OR (CI)	Wheeze: *p*-value	Current Asthma: OR (CI)	Current Asthma: *p*-value
PFOA	1.13 (0.99, 1.29)	0.06	1.01 (0.85, 1.20)	0.92	1.06 (0.91, 1.25)	0.45
PFOS	0.86 (0.75, 0.98)	0.03	0.87 (0.73, 1.03)	0.10	0.89 (0.75, 1.05)	0.17
PFNA	1.01 (0.91, 1.13)	0.79	1.01 (0.86, 1.18)	0.94	1.03 (0.87, 1.23)	0.71
PFHS	0.99 (0.91, 1.08)	0.81	1.00 (0.92, 1.09)	0.98	1.02 (0.91, 1.15)	0.73

*$p<0.05$. All models adjusted for sex, smoking, age, race/ethnicity, survey cycle, poverty income ratio, and health insurance.

Multiple imputation of missing data (i.e. 10 imputations) were conducted as per Berglund et al, 2010.

Table S3. Odds Ratios of 'Ever Asthma', 'Wheeze in Past Year', and 'Current Asthma' for values above the limit of detection of EPAH, MPAH, PFDE, and PFSA among children ages 12-19 (n = 1,877).

PFC	Ever Asthma: OR (CI)	Ever Asthma: *p*-value	Wheeze: OR (CI)	Wheeze: *p*-value	Current Asthma: OR (CI)	Current Asthma: *p*-value
EPAH	0.93 (0.65, 1.34)	0.70	1.19 (0.75, 1.88)	0.46	0.97 (0.58, 1.61)	0.91
MPAH	1.33 (0.65, 2.73)	0.44	1.62 (0.61, 4.27)	0.33	1.10 (0.37, 3.31)	0.87
PFDE	0.87 (0.60, 1.28)	0.49	0.97 (0.62, 1.50)	0.89	0.96 (0.59, 1.56)	0.88
PFSA	0.92 (0.68, 1.27)	0.63	0.98 (0.64, 1.50)	0.94	0.91 (0.63, 1.32)	0.62

All models adjusted for sex, smoking, age, race/ethnicity, survey cycle, poverty income ratio, and health insurance.

Reference

Berglund PA. 2010. An Introduction to Multiple Imputation of Complex Sample Data using SAS®v9.2. In: SAS Global Forum 2010.
